# Endovascular Thrombectomy Following Acute Ischemic Stroke: A Single-Center Case Series and Critical Review of the Literature

**DOI:** 10.3390/brainsci3020521

**Published:** 2013-04-12

**Authors:** Eric Sussman, Christopher Kellner, Michael McDowell, Peter Yang, Eric Nelson, Sophie Greenberg, Daniel Sahlein, Sean Lavine, Philip Meyers, E. Sander Connolly

**Affiliations:** Department of Neurological Surgery, Columbia University Medical Center, 710 West 168th Street, New York, NY 10032, USA; E-Mails: esuss11@gmail.com (E.S.); christopher.kellner@gmail.com (C.K.); mmm2292@columbia.edu (M.M.); peterhyy@gmail.com (P.Y.); ean2120@columbia.edu (E.N.); sophieagreenberg@gmail.com (S.G.); ds3096@columbia.edu (D.S.); sl2081@columbia.edu (S.L.); pmm2002@columbia.edu (P.M.)

**Keywords:** acute ischemic stroke, endovascular, mechanical thrombectomy

## Abstract

Acute ischemic stroke (AIS) due to thrombo-embolic occlusion in the cerebral vasculature is a major cause of morbidity and mortality in the United States and throughout the world. Although the prognosis is poor for many patients with AIS, a variety of strategies and devices are now available for achieving recanalization in patients with this disease. Here, we review the treatment options for cerebrovascular thromboembolic occlusion with a focus on the evolution of strategies and devices that are utilized for achieving endovascular clot extraction. In order to demonstrate the progression of this treatment strategy over the past decade, we will also present a single-center case series of AIS patients treated with endovascular thrombectomy.

## 1. Introduction

Stroke is the fourth leading cause of death in the United States and is also one of the leading causes of long-term disability [1,2,3,4,5,6,7,8,9]. Despite increased public awareness and widespread efforts to address risk factors for this devastating disease, there are almost 800,000 new cases of stroke per year [2,10]. Ischemia is the most common underlying mechanism, accounting for 87% of all strokes [4,11]. 

Prior to the use of thrombolytic agents in patients with acute ischemic stroke (AIS), treatment options were limited. Over the past two decades, AIS management has evolved tremendously, and there are now a variety of drugs and devices available. Here, we review the evolution of treatment modalities for AIS, beginning with a brief review of intravenous thrombolysis, followed by an in-depth look at endovascular techniques. We will focus on the evolution of strategies and devices that are utilized for achieving endovascular clot extraction. In order to demonstrate the progression of this treatment strategy over the past decade, we will also present a single-center case series of AIS patients treated with endovascular thrombectomy between January 2006 and December 2012. 

### 1.1. Intra-Venous Thrombolysis

In 1996—based on the results of the National Institute of Neurological Disorders and Stroke (NINDS) rtPA Stroke Study Group trial [12,13]—the U.S. Food and Drug Administration (FDA) approved intravenous (IV) recombinant tissue plasminogen activator (rtPA) for the treatment of AIS. In the decade that followed, clinical data reaffirmed a clear benefit of IV rtPA in patients with AIS [10,12,13,14,15,16,17,18,19,20,21]. Nevertheless, it is estimated that only 1.8% to 2.1% of AIS patients receive IV thrombolysis (IVT) [10,22,23,24], which is at least partially attributable to the narrow three-hour time window for drug administration. Based on the results of the ECASS-3 trial published in 2008, the window of eligibility for IV rtPA was expanded to 4.5 h for a subset of AIS patients [19,25,26,27,28,29,30,31], and this is likely to increase the number of AIS patients able to be treated with IVT. However, even if time was not an exclusion criteria, only approximately 30% of AIS patients would be eligible for IV rtPA [32,33]. Furthermore, among the subset of patients who satisfy all eligibility criteria, only a minority of patients achieves an observable clinical benefit from treatment with IVT. More specifically, the NINDS trial of IV rtPA in AIS demonstrated only a 12% absolute increase in favorable functional outcomes (and showed no mortality benefit) in AIS patients treated with IV rtPA *versus* those treated with placebo [13,34,35]. In addition, due to its potent fibrinolytic effect, rtPA is associated with an increased risk of bleeding events, including systemic hemorrhage and intracerebral hemorrhage (ICH), and this risk outweighs the potential benefits of the drug beyond the 3- to 4.5-h window. In light of these limitations, alternative therapies have been devised for patients with AIS. There are a number of novel drugs in the translational pipeline or in clinical trials [1,3,5,6,7,8,9,36], but IV rtPA is currently the only FDA-approved fibrinolytic agent with an AIS indication. 

### 1.2. Intra-Arterial Fibrinolysis

The intra-arterial administration of fibrinolytic agents obviates some of the limitations of the systemic intravenous route. The primary advantage of IA fibrinolysis (IAF) is the ability to administer the fibrinolytic agent directly into the occluded vessel, thereby maximizing the amount of drug that reaches the target vessels, while simultaneously minimizing systemic fibrinolysis. In this way, the risk of non-target hemorrhage may theoretically be reduced [10,37,38]. However, IAF procedures in general have some unique disadvantages, including increased time required for treatment initialization and increased procedural complexity and costs, as well as an inherent risk of blood vessel injury associated with an endovascular procedure [10].

The safety and efficacy of IAF, using prourokinase (Pro-lyse) as the fibrinolytic agent, was demonstrated in the phase II Pro-lyse in Acute Cerebral Thromboembolism (PROACT-I) trial [4,39], as well as in the phase III PROACT-II trial [12]. In each of these trials, patients were randomized to either an experimental group (IA prourokinase plus low-dose IV heparin) or a control group (low-dose IV heparin alone), and treatment was administered within 6 h of symptom onset. Those patients who received IA prourokinase had significantly higher rates of recanalization and of a favorable functional outcome (defined as mRS ≤ 2) compared with controls. The rate of symptomatic ICH was higher in the experimental group than in the control group. Despite this, there was no difference in mortality between the groups. Although the rate of favorable functional outcome was higher in patients treated with IA prourokinase in the PROACT-II trial (40%) than in those patients treated with IV rtPA in the NINDS trial (31%), it is impossible to make any definitive statements regarding the relative efficacy of IVT and IAF, as there have not been any randomized controlled trials directly comparing the two. Furthermore, although the literature strongly suggests a higher recanalization rate in patients treated with IAF compared with those treated with IVT [12,14,16,18,20,21], there remains a significant proportion of AIS patients for whom fibrinolytic therapy alone, regardless of the route of delivery, is inadequate at recanalizing the occluded vessels.

Early studies of AIS intervention showed that time to treatment is an important indicator of patient outcome [12]. Because of delays inherent in catheter-based intervention, attention turned to rapid administration of IV fibrinolytic therapy during transit to the angiography suite for endovascular intervention. The theoretical benefit of such a “bridging protocol” is that it preserves all of the advantages of IAF without the detraction of treatment delay as the resources for an endovascular procedure are mobilized. The Emergency Management of Stroke (EMS) Bridging Trial confirmed the feasibility of a combined IV and IA approach to fibrinolysis. This trial randomized AIS patients to either an experimental bridging protocol group (IVT followed by IAF) or a control group (IV-placebo followed by IAF) and demonstrated an increased rate of recanalization with the bridging protocol, but no difference in functional outcomes [23]. Bridging protocols have been further investigated in the Interventional Management of Stroke (IMS) studies, a series of three prospective trials of IAF in patients with persistent occlusion following IV rtPA treatment [27,28,30]. Patients enrolled in IMS-I and -II had significantly better functional outcomes than placebo-treated patients from the NINDS trial and marginally better outcomes when compared to NINDS patients who received IV rtPA. However, the recently published IMS-III trial demonstrated no improvement in outcomes in patients treated with endovascular modalities following IVT (as will be discussed below) [40].

PROACT-II demonstrated the benefit of IAF by administration of pro-urokinase at the proximal margin of the occlusive thromboembolus alone. An additional advantage of the intra-arterial route of fibrinolytic administration is that it allows for direct manipulation of the occluding thrombus. Mechanical clot disruption is most easily achieved by repeatedly passing the endovascular guidewire across the site of obstruction, thereby macerating the clot and increasing the surface area upon which the fibrinolytic agent can have its effect. A number of novel strategies and devices have been developed to facilitate this process of mechanical clot disruption. For instance, continuous transcranial Doppler ultrasound has been utilized to direct ultrasonic energy towards the occluded vessel and a sonographically-activated microcatheter—the EKOS micro-infusion catheter—has been developed to focus ultrasonic energy into the immediate vicinity of the occlusion. In order to evaluate the efficacy of mechanical clot disruption as an adjunct to IAF, a subset of IMS-II patients were treated with an EKOS Primo Micro-Infusion Catheter at the time of IAF administration. Although patients treated with the sonographically-activated EKOS catheter had a numerically higher rate of recanalization as compared with a similar cohort of patients who received IAF alone, this difference did not reach statistical significance [26]. Laser thrombolysis instruments have also been developed, including the LaTIS (Spectanetics, Colorado Springs, CO) and Endovascular Photo-Acoustic Recanalization (EPAR, Endovasix, Belmont, CA), but trials of these devices have not demonstrated a definitive clinical benefit [10,22]. 

Balloon angioplasty has been used to treat coronary and other peripheral arterial occlusions for many years. Similarly, a thromboembolic occlusion of a cerebral artery can be directly manipulated using balloon angioplasty. Although angioplasty has long been used non-acutely in patients with cerebral vascular stenosis [25], the first reports of using this technique in the setting of acute stroke are relatively more recent [32]. Nevertheless, angioplasty for the treatment of AIS has been shown to be a safe strategy with high rates of recanalization [34,35]. Therefore, angioplasty has become an increasingly common technique in multimodal endovascular approaches to patients with AIS, particularly those patients with refractory vessel occlusion following multiple treatment attempts. The intention of angioplasty is to radially displace or fragment the occlusive thrombus towards the periphery of the blood vessel, thereby reestablishing blood flow through the site of occlusion. Similarly, stent deployment, either alone or in conjunction with balloon angioplasty, has been shown to be a highly effective means of achieving flow restoration in patients with AIS. In a meta-analysis of studies in which patients received cerebrovascular stents for the treatment of AIS, successful recanalization was achieved in 113 of 127 patients (89%), while hemorrhagic complications occurred in 26 patients (20.5%), 12 (9.4%) of which were symptomatic [41]. In the only prospective trial included in this meta-analysis—the SARIS Trial [36]—successful recanalization was achieved in 100% of the 20 enrolled patients using the Wingspan Stent (Boston Scientific, Natick, MA), with only one (5%) symptomatic and two (10%) asymptomatic ICHs. More recently, 105 patients with AIS who were either ineligible for or failed to respond to IV rtPA were assigned to receive either balloon angioplasty with stent placement or no further treatment [42]. Those patients who underwent balloon angioplasty with stent placement had significantly higher rates of recanalization and favorable functional outcome (mRS ≤ 2). Although the rate of recanalization seems to be higher with stenting than with alternative endovascular therapies for AIS, with a similar rate of procedure-related complications, the use of stents in patients with AIS has been limited by concerns about the need for dual anti-platelet therapy, the increased risk of hemorrhage and possible in-stent stenosis.

### 1.3. Endovascular Thrombectomy

In contrast to IVT or IAF, in which fibrinolytic agents are utilized to expedite the process of clot dissolution, endovascular thrombectomy aims to extract the clot from the occluded vessel. There are a number of advantages of mechanical thrombectomy, including rapid flow restoration, a decreased risk of clot fragmentation and distal embolization and increased rates of recanalization in the context of platelet-rich clots, which are more resistant to fibrinolysis [37,38]. Furthermore, by allowing for a reduction in the required dose of fibrinolytic, mechanical thrombectomy may be associated with a reduced risk of ICH, therefore justifying endovascular intervention at more distant time points after AIS onset.

There are a multitude of devices and strategies that have been developed for mechanical thrombectomy in the cerebral vasculature. Discussion of these devices is organized according to the location the device is deployed relative to the site of occlusion: (1) distal to the site of occlusion, (2) proximal to the occlusion or (3) at the site of the occlusion. As described above, one of the primary advantages of an intra-arterial approach to AIS management is the opportunity to administer multiple endovascular therapies in order to achieve successful flow restoration. Many neurointerventionalists have adopted a multimodal approach, combining mechanical thrombectomy with IAF and/or angioplasty/stenting and the safety and efficacy of such a multi-modal approach will also be discussed.

#### 1.3.1. Distally Deployed Devices

**Figure 1:**
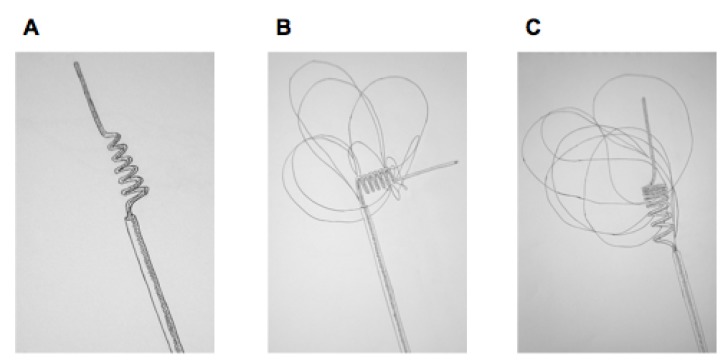
(**A**) First generation Merci retriever; (**B**) second generation; (**C**) third generation.

Distally deployed devices are passed beyond the site of the occlusion prior to device deployment. and the clot is subsequently engaged and pulled out of the vasculature along with the endovascular instrumentation. There are a number of such devices that have been developed specifically for the cerebral vasculature. The most widely used—and the only such device with FDA-approval for use in AIS patients—is the Merci Retriever (Concentric Medical, Mountain View, CA). First-generation Merci retrievers (X-Series) consist of a distally tapered helix of nitinol wire, which engages the thrombus in a “cork-screw” fashion. Second-generation devices (L-Series) consist of a non-tapered helix of nitinol wire that lies at a 90°-angle relative to the proximal portion of the device and also contains a web of arcading filaments extending distally from the wire. Third-generation devices (V-Series) consist of a non-tapered helix of nitinol wire with variably spaced coils, as well as a distal web of arcading filaments (Figure 1).

The safety and efficacy of mechanical thrombectomy using the Merci retriever was demonstrated in a phase I trial published in 2004 [39] and subsequently confirmed in a phase II trial published in 2005 [43]. In the phase I trial, 28 patients with a contraindication to IV rtPA were treated with the Merci device within 8 h of AIS symptom onset. Successful recanalization was achieved in 12 (43%) of these patients when the Merci device was used alone and in 18 (64%) patients when adjunctive IA rtPA was administered. There was one procedure-related complication (unintended detachment of the tip of the Merci retriever) that was rectified without clinical consequence and 12 ICHs, all of which were asymptomatic. In the similarly designed phase II trial, recanalization was achieved in 68/141 (48%) patients and in 85 patients (60%) when adjunctive rtPA was used. Clinically significant procedural-related complications occurred in 10 patients (7.1%), and symptomatic ICH occurred in 11 patients (7.8%). There was no difference in the rate of hemorrhage between those patients who were treated with the Merci device alone *versus* those treated with adjuvant IAF. This trial confirmed the safety and efficacy of the Merci retriever for mechanical thrombectomy in AIS, leading to the 2004 FDA-approval of this device for patients who fail or are ineligible for IV rtPA.

An additional multi-center, prospective, single-arm trial of mechanical thrombectomy with the Merci retriever device—The Multi-MERCI trial [13,44]—was similar to the phase I and II trials described above with the notable exception that patients with persistent large vessel occlusion after IV rtPA treatment were eligible for inclusion. In addition, the Multi-MERCI trial utilized the newer second-generation Merci devices, in contrast to the phase I and II trials discussed above, in which the first-generation devices were used. Published in 2008, this trial demonstrated successful recanalization in 75/131 patients (57.3%), with clinically significant procedure-related complications occurring in nine patients (5.5%) and symptomatic ICH occurring in 16 patients (9.8%). Furthermore, the rate of successful recanalization increased to 69.5% (91/131 patients) following adjunctive treatment with IA rtPA and/or additional attempts at clot extraction using the older-generation Merci devices. Importantly, despite the inclusion of patients who had received IV rtPA prior to endovascular intervention, there was no significant increase in the rate of ICH or procedural-related complications as compared with the phase I- and II trials described above, therefore suggesting that the Merci retrieval device may be safely utilized in AIS patients with persistent occlusion after IV rtPA. 

A number of other distally deployed devices have been developed and successfully utilized to perform mechanical thrombectomy in patients with AIS, including the Neuronet device (Guidant, Santa Clara, CA) [10,13,15,17,19,45], the Catch thrombectomy device (Balt, Montmorency, France) [24,46], the Phenox Clot Retriever (Phenox, Bochum, Germany) [19,29,31,47], the Attracter-18 device (Target Therapeutics, Fremont, CA) [48] and the Alligator Retrieval Device [49,50]. However, the safety and efficacy of these devices has not been firmly established in large prospective trials, as in the case of the Merci devices, and therefore, the Merci retriever remains the only distally deployed device with FDA approval for use in AIS patients.

#### 1.3.2. Proximally Deployed Devices

**Figure 2:**
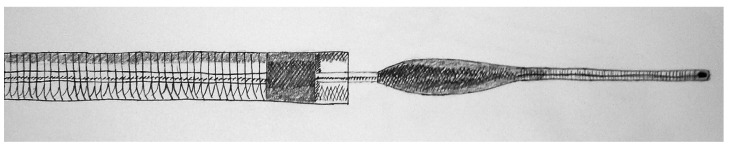
Penumbra System, consisting of both a thromboaspiration suction device and a separator.

With proximally deployed devices, the clot is grasped or aspirated from the proximal vasculature. The Penumbra Thromboaspiration System (Penumbra, Alameda, CA) is the most widely used and the only such device with FDA approval for use in AIS patients. The Penumbra System consists of two components: a reperfusion microcatheter attached to a suction pump and a separator, which is a cone-shaped guidewire that serves to clear clot fragments from the distal end of the microcatheter in order to maintain continuous suction (Figure 2). The theoretic advantage of a thromboaspiration strategy is a reduced risk of clot fragmentation and distal embolization.

The safety and efficacy of the Penumbra System was evaluated in a phase I trial, in which 23 patients presenting within 8 h of AIS symptom onset were enrolled [51]. Of the 23 enrolled patients, only 20 (87%) were treated with the Penumbra System, due to difficulty navigating the thromboaspiration device through the cerebral vasculature in three of the patients. Successful recanalization (defined as a thrombolysis in myocardial infarction [TIMI] score of 2 or 3) was achieved in 100% of patients treated with the Penumbra System alone, although nine patients received adjunctive IA rtPA in order to achieve complete (TIMI 3) flow restoration after thromboaspiration. Eight patients experienced ICH, two of which were symptomatic. Importantly, seven of the eight cases of ICH occurred in patients treated with adjunctive IA rtPA. The Penumbra Pivotal Stroke Trial was a phase II trial performed in 2009 to further evaluate the safety and efficacy of the Penumbra System in AIS [52]. In this prospective, multi-center, single-arm trial, 125 patients were treated with the Penumbra System. Successful recanalization was achieved in 102 patients (81.6%), with 16 patients (12.8%) experiencing procedure-related complications, three (2.4%) of which were classified as severe. Additionally, 35 patients (28%) had post-procedure hemorrhage, and 14 (11.2%) of these were symptomatic. The initial post-market performance of the Penumbra System was also evaluated in the POST Trial [53], a retrospective review of 157 consecutive patients treated with the Penumbra System at seven international centers. In this series, TIMI 2- or 3-recanalization was achieved in 87% of patients, with nine patients (5.7%) experiencing serious procedure-related adverse events. 

The Neurojet (Possis Medical, Minneapolis, MN) is another thromboaspiration device developed specifically for use in the cerebral vasculature, but the pilot study was discontinued, due to a high rate of endovascular injury and difficulties navigating the cerebral vasculature [10,54]. In addition, a novel Penumbra separator device—the 3D separator—is currently being evaluated in two trials: the 3D Separator Trial comparing the Penumbra system with the 3D separator to the Penumbra system alone [55] and the THERAPY Trial assessing the safety and efficacy of the Penumbra 3D separator as an adjunct to IV rtPA therapy [56].

#### 1.3.3. Intra-Clot Deployed Devices

**Figure 3:**
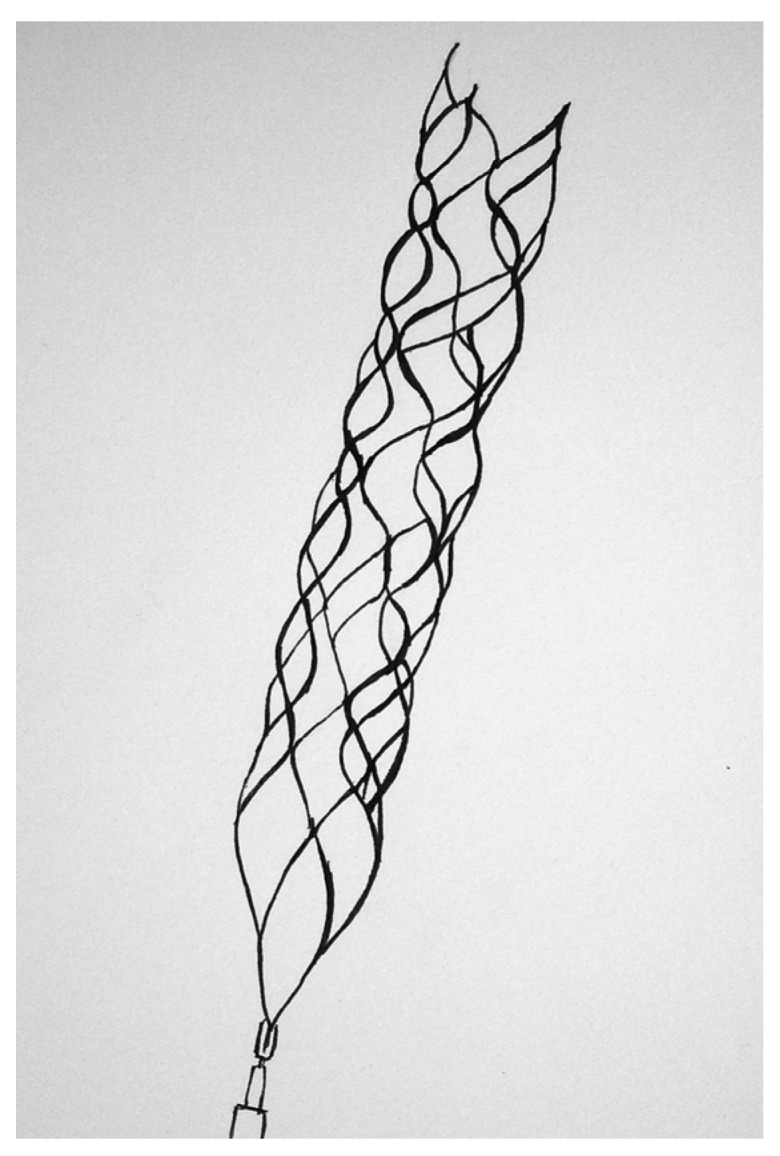
Solitaire flow restoration device.

As described previously, cerebrovascular stenting leads to a high rate of recanalization in patients with AIS, but more widespread implementation of this technique has been limited by concerns about the need for dual anti-platelet therapy, the increased risk of hemorrhage and possible in-stent stenosis. In order to circumvent some of these limitations, a novel technique has been utilized in which stents are partially deployed and subsequently removed with the clot embedded within the struts of the stent. This technique of using reversible stents (RS), or stentrievers, combines the advantages of angioplasty and stenting with those of mechanical clot extraction. The two most commonly used stentrievers are the Solitaire Flow Restoration Device (ev3, Plymouth, MN) (Figure 3) and the Trevo Retriever (Concentric Medical, Mountain View, CA). The safety and efficacy of the Solitaire stentriever was compared with that of the Merci device in a randomized, parallel-group non-inferiority study—the Solitaire with intention for thrombectomy (SWIFT) trial. Due to overwhelming superiority (*p* = 0.0001) of the Solitaire device at the interim analysis, the SWIFT Trial was terminated early in 2011, after only 126 of the anticipated 250 patients were enrolled [57], and the Solitaire device has since been FDA approved with an indication for clot retrieval in AIS. The efficacy of the Trevo Retriever was also assessed in The Thrombectomy Revascularization of Large Vessel Occlusions in Acute Ischemic Stroke (TREVO-2) Trial [58], an open-label randomized controlled trial that compared the Trevo Retriever to the Merci device. Similar to the results of the SWIFT trial, the TREVO-2 Trial found that the Trevo Retriever had superior efficacy as compared with the Merci device. In a 2012 prospective trial comparing the Trevo Retriever and the Solitaire device, the Trevo Retriever was associated with a higher rate of recanalization [59]; however, this trial was small (*n* = 33) and not randomized. Without an adequately powered randomized controlled trial, it is impossible to draw a definitive conclusion regarding the relative efficacy of the Trevo and Solitaire devices.

### 1.4. Combined Approaches

In order to evaluate the pragmatic multimodal approach to AIS intervention that is typically employed in clinical practice, several recent studies have compared the efficacy of a combined approach—consisting of IVT, as well as one or more IA modalities—to IVT alone. The SYNTHESIS Expansion Trial randomized 362 AIS patients presenting within 4.5 h of symptom onset to either IVT or IA intervention (either IAF, mechanical clot disruption or embolectomy or a combination of these approaches). The results of this trial fail to demonstrate superiority of an endovascular approach as compared with IVT [60]. Another trial mentioned above—IMS-III—randomly assigned eligible patients who had received IV t-PA to further endovascular treatment or to no additional intervention. This trial was terminated on the basis of futility of further endovascular intervention after 656 of the planned 900 participants had been enrolled [40]. Importantly, in both of these trials, the time to intervention was longer in the IA arm than in the IVT arm, which may have contributed to the lack of superior clinical benefit of an endovascular approach. Although subgroup analyses of these trials suggested that the lack of clinical benefit was not due to endovascular treatment delays, future trials should strive to minimize this discrepancy in time to treatment.

The MR RESCUE Trial assessed the utility of pretreatment neuroimaging (either CT or MRI) for identifying patients with a favorable penumbral pattern who are most likely to benefit from endovascular intervention, either as the initial treatment or after failed recanalization with IV tPA (provided that pretreatment neuroimaging demonstrated substantial salvageable tissue). The results failed to demonstrate that pretreatment brain imaging allowed for identification of patients who would differentially benefit from further endovascular intervention. In addition, similar to the SYNTHESIS Expansion and IMS-III trials described above, the MR RESCUE Trial failed to demonstrate superiority of endovascular intervention over standard medical therapy with IV tPA [61].

## 2. Methods

This case series includes all patients who underwent endovascular mechanical thrombectomy for AIS between January 2006 and December 2012 at Columbia University Medical Center. Monthly case logs were obtained from the Department of Neurosurgery, and all patients with AIS were identified using *International Classification of Diseases, Clinical Modification, 9th Revision (ICD-9)* primary diagnosis codes 433–434.91. Among all patients with AIS as identified by ICD-9 codes, those patients who were treated via endovascular intervention (endovascular thrombolysis, mechanical thrombectomy or angioplasty/stenting) were identified using current procedural terminology (CPT) codes. Patients with venous sinus thromboses were excluded, as were patients who were undergoing non-acute intervention for recurrent transient ischemic symptoms.

For each patient who underwent mechanical thrombectomy, medical records were retrospectively reviewed, including demographic, admission, laboratory, imaging, procedural, discharge and follow-up data. Pre-procedural National Institute of Health Stroke Scale (NIHSS) scores were obtained from the admission exam documentation. The degree of post-procedural recanalization was classified as “incomplete”, “partial” or “complete/nearly complete” based on the thrombolysis in cerebral infarction (TICI) score (Table 1). The TICI score was either reported by the attending physician in the procedural note or else determined by retrospective review of intra-operative angiographic images. Information regarding procedural complications—including vessel dissection or rupture, groin hematoma, device malfunction or clot embolization to a previously non-occluded vessel—was obtained from the procedural notes. All imaging scans obtained following the procedure were reviewed in order to identify post-procedural hemorrhages, and for those patients who were found to have radiographic evidence of hemorrhage, progress notes were reviewed to further classify these as symptomatic (*i.e.*, coinciding with a deterioration in neurological exam) or asymptomatic (*i.e.*, no deterioration in neurological exam coincident with the radiographic finding of hemorrhage). The documented discharge exam was used to calculate discharge mRS for each patient: “good outcome” was defined as mRS ≤ 2. 

**Table 1:** Description of thrombolysis in cerebral infarction (TICI) score and corresponding degree of recanalization used in this case series.

Score	TICI Recanalization	Degree of Recanalization
**0**	No perfusion distal to the occlusion	Incomplete
**1**	Perfusion past the site of occlusion, but no significant distal branch filling	
**2a**	Incomplete (<50%) distal branch filling	Partial
**2b**	Incomplete (>50%) distal branch filling	Complete or nearly complete
**3**	Full perfusion with filling of all distal branches	

## 3. Results

A total of 128 patients were identified with ICD-9 codes consistent with AIS, as well as CPT codes consistent with an endovascular intervention. After reviewing the medical records, four patients were excluded, due to primary diagnoses of venous sinus thrombosis, and seven patients were excluded, due to the fact that the indication for endovascular intervention was recurrent chronic ischemic symptoms, rather than AIS. Among the remaining 117 patients, 84 underwent mechanical thrombectomy, either alone or as part of a multi-modal endovascular approach (*i.e.*, in conjunction with IAF and/or angioplasty/stenting), and 33 patients underwent primary IAF.

### 3.1. Pre-Procedural Data

Of the 84 patients who underwent mechanical thrombectomy and were included in the analysis, the average age was 64.2 (SD 16.7). Seventy-four patients (88.1%) had anterior circulation occlusions, and 10 patients (11.9%) had occlusions in the posterior circulation. More specifically, the occlusion was in the internal carotid artery (ICA) in 29/84 patients (34.5%), in the middle cerebral artery (MCA) in 43/84 (51.2%), in the anterior cerebral artery (ACA) in 1/84 (1.2%), in the vertebral artery in 2/84 (2.4%) and in the basilar artery in 9/84 (10.7%). Forty-nine of the 84 patients (58.3%) had occlusions in the left-side circulation and 25/84 (29.8%) had occlusions in the right-side circulation.

Thirty-one of the 84 patients (36.9%) received IV rtPA prior to endovascular intervention, and the average time between symptom onset (and time of last known normal) and IV rtPA administration was 120 min (standard deviation [SD] 38.4 min). The average pre-operative National Institute of Health Stroke Scale (NIHSS) score among all patients who underwent mechanical thrombectomy was 19.7 (SD 6.8). Among patients who received IV rtPA prior to mechanical thrombectomy, the average pre-operative NIHSS score was 19.2 (SD 5.2), *versus* 20.0 (SD 7.7) in those who did not receive IV rtPA. 

### 3.2. Procedural Data

**Figure 4:**
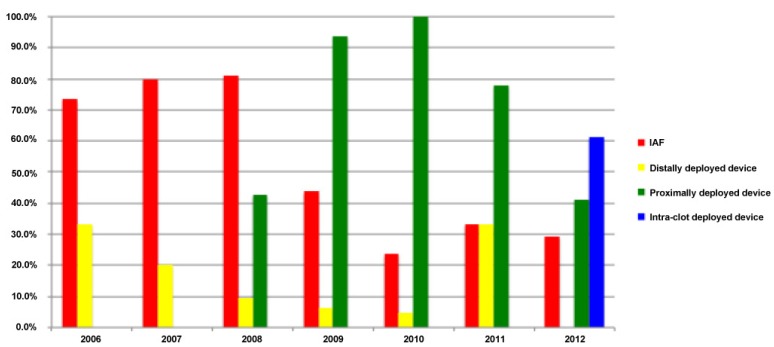
Endovascular treatment modalities used by year (as a percentage of all endovascular interventions for acute ischemic stroke (AIS)).

Sixteen patients (18.8%) were treated with a distally deployed device (15 with the Merci Retriever and one with the Attractor-18), 64 (75.3%) were treated with a proximally deployed device (the Penumbra Thromboaspiration System in all cases) and 11 (12.9%) were treated with an intra-clot deployed device (10 with the Solitaire Stentriever and one with the Trevo Retriever). In seven of the 16 cases in which the distally deployed device was used, it was done so only after a failed attempt at recanalization using the Penumbra aspiration system. Twenty-eight of 84 patients (33.3%) received IA-rtPA in addition to mechanical thrombectomy, including 3/9 (33.3%) of those treated with a distally deployed device, 20/57 (35.1%) of those treated with a proximally deployed device and 5/11 (45.5%) of those treated with an intra-clot deployed device. None of the seven patients treated with both distally and proximally deployed devices received IAF. Of the 28 patients who received IAF, 12 also received IV rtPA prior to endovascular intervention. In addition, 6/84 patients (7.1%) received balloon angioplasty, and intravascular stents were placed in four of these cases. For a graphic depiction of the proportion of AIS patients who were treated with each of the different endovascular modalities during each of the seven years of this case series, see Figure 4.

The average procedure duration for all patients who underwent mechanical thrombectomy was 154 min (SD 56): 180 min (SD 30) when a distally deployed device was used, 150 min (SD 62) with proximally deployed devices, 162 min (SD 45) with intra-clot deployed devices and 168 min (SD 57) with combined distally and proximally deployed devices. Among those patients who underwent mechanical thrombectomy with adjunctive IAF, the average procedure duration was 169 min (SD 44), *versus* 149 min (SD 60) among those who did not receive adjunctive IAF.

Complete or nearly complete recanalization (TICI 2b or 3) was achieved in 40.5% of all patients who underwent mechanical thrombectomy. When a distally deployed device was used alone, the rate of complete or nearly complete recanalization was 0.0%, while 40.4% of patients achieved this degree of recanalization with a proximally deployed device, 81.8% with an intra-clot deployed device and 28.6% with combined distally and proximally deployed devices (Table 2). In addition, the rate of complete or nearly complete recanalization was 42.9% among those patients who received IA-rtPA and 40.4% among those who did not. 

**Table 2:** Degree of recanalization stratified by type of device used.

Degree of Recanalization		Number	Percentage
**Distally deployed device (*n* = 9)**			
	Incomplete	5/9	55.6%
	Partial	4/9	44.4%
	Complete/nearly complete	0/9	0.0%
**Proximally deployed device (*n* = 57)**			
	Incomplete	18/57	31.6%
	Partial	16/57	28.1%
	Complete/nearly complete	23/57	40.4%
**Combined proximally & distally deployed devices (*n* = 7)**			
	Incomplete	3/7	42.9%
	Partial	2/7	28.6%
	Complete/nearly complete	2/7	28.6%
**Intra-clot deployed device (*n* = 11)**			
	Incomplete	2/11	18.2%
	Partial	0/11	0.0%
	Complete/nearly complete	9/11	81.8%

Procedure-related complications occurred in 6 patients (7.1%), including four intra-operative hemorrhages (one of which was caused by vessel rupture), one groin hematoma and one device malfunction. In three of the four cases complicated by intra-operative hemorrhage (including the one complicated by vessel rupture), a proximally deployed device was used, while a distally deployed device was utilized in the fourth case. The device malfunction involved an unintended detachment of a Solitaire stentriever device tip, which was left *in situ* without clinical consequence. In addition, clot fragmentation and distal embolization occurred in 10/84 (11.9%) cases, (2/9 cases with distally deployed devices, 7/57 cases with proximally deployed devices and 1/11 cases utilizing an intra-clot deployed device), although this resulted in occlusion of a previously non-occluded vessel in only 3 cases.

### 3.3. Post-Procedural Data

Reperfusion hemorrhage was noted on post-procedural radiographic imaging in 44/84 patients (52.4%), and 13 (15.5%) of these were symptomatic, (Table 3). A good outcome (mRS ≤ 2) was observed in 14/84 patients (16.7%), while in hospital mortality occurred in 25/84 patients (29.8%), (Table 4).

**Table 3:** Rate of reperfusion hemorrhage (total and symptomatic) stratified by type of device used and by adjunctive administration of fibrinolytic therapy. IVT, IV thrombolysis; IAF, IA fibrinolysis.

	Total Reperfusion Hemorrhage		Symptomatic Reperfusion Hemorrhage	
**Total**	44/84	52.4%	13/84	15.5%
**With IVT**	18/31	58.1%	7/31	22.6%
**Without IVT**	26/53	49.1%	6/53	11.3%
**With IAF**	14/27	51.9%	5/27	18.5%
**Without IAF**	30/57	52.6%	8/57	14.0%
**Distally deployed device**	5/9	55.6%	1/9	11.1%
**Proximally deployed device**	29/57	50.9%	9/57	15.8%
**Combined proximally and distally deployed devices**	3/7	42.9%	1/7	14.3%
**Intra-clot deployed device**	7/11	63.6%	2/11	18.2%

**Table 4:** Functional outcome stratified by type of device used and by adjunctive administration of fibrinolytic therapy.

	Good Outcome (mRS ≤ 2)		Death (mRS = 6)	
**Total**	14/84	16.7%	25/84	29.8%
**With IVT**	3/31	9.7%	10/31	32.3%
**Without IVT**	11/53	20.8%	15/53	28.3%
**With IAF**	7/27	25.9%	8/27	29.6%
**Without IAF**	7/57	12.3%	17/57	29.8%
**Distally deployed device**	1/9	11.1%	2/9	22.2%
**Proximally deployed device**	8/57	14.0%	18/57	31.6%
**Combined proximally and distally deployed devices**	1/7	14.3%	2/7	28.6%
**Intra-clot deployed device**	4/11	36.4%	3/11	27.3%

## 4. Discussion

As demonstrated by this case series, there are a multitude of different strategies and devices available for the endovascular management of AIS, and the patterns of AIS management have been continuously changing over the past decade. In 2006 and 2007, a majority of AIS patients who underwent endovascular intervention at our institution were treated with IAF. Furthermore, greater than 50% of these patients did not undergo mechanical thrombectomy as an adjunct to IAF, despite the fact that the Merci retriever was FDA approved for use in this population. This practice pattern may be a reflection of the available data at the time, which suggested that the rates of recanalization and of favorable functional outcome were not significantly different in patients treated with IAF *versus* those treated with the Merci retriever [4,12,39,43]. In those patients in which mechanical thrombectomy was attempted, however, a distally deployed device (most commonly the Merci retriever) was used in all cases.

**Figure 5:**
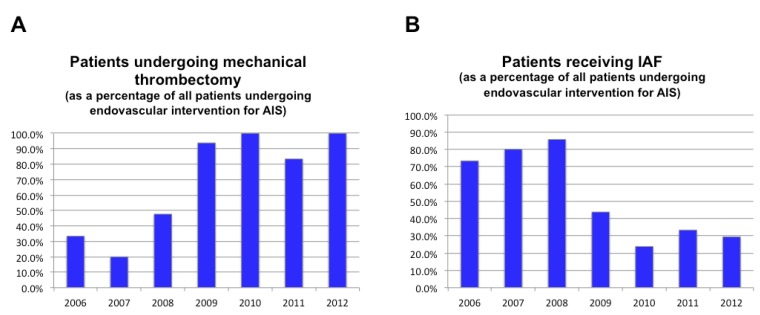
(**A**) Percentage of AIS patients treated by endovascular intervention each year who underwent mechanical thrombectomy. (**B**) Percentage AIS patients treated by endovascular intervention each year who received IAF.

Beginning in 2008, we observed an increase in the proportion of endovascularly-managed AIS patients who were treated with mechanical thrombectomy. This shift coincided with increasing evidence in the literature, suggesting higher rates of recanalization with proximally deployed devices [51,52,53]. It is therefore not surprising that in 2008, a proximal-approach to mechanical thrombectomy began to supplant the distal-approach at our institution. In fact, due to the growing evidence of improved rates of recanalization with the Penumbra aspiration device, this management technique became the status quo for AIS patients at our institution by 2009. Interestingly, between 2008 and 2009, we also observed a sharp decrease in percentage of patients treated with IAF, likely reflective of the increased recanalization rates that were achievable with the Penumbra aspiration device alone (Figure 5). 

Practice patterns at our institution underwent another shift in 2012, when the use of retrievable stents became the preferred method of mechanical thrombectomy. This transition occurred following the publication of the results of the SWIFT Trial discussed previously, which demonstrated a rate of recanalization with Solitaire stentriever that was greater than that previously reported for any other available device or strategy [57]. In fact, beginning in April 2012, every patient who underwent endovascular intervention for AIS at our institution was treated with a retrievable stent device.

## 5. Conclusions

The intention of this case series was not to evaluate the relative efficacy of the various strategies and devices that are currently available, but rather to demonstrate the rapid progress in the field of AIS management and to explore the factors that drive this evolution. There are currently many strategies and devices available, and these may be combined in a seemingly limitless number of ways. While a variety of factors are considered when determining how to treat any individual patient, it is evident from this case series that practice patterns are largely driven by the contemporary literature regarding the relative efficacy of the available strategies and devices.

As this dynamic and growing field continues to develop, there will undoubtedly be a plethora of new devices and strategies that become available. As such, there will be a continual need for investigation and re-evaluation in order to establish current evidence-based practice guidelines. 

## References

[1] Haley E.C., Lyden P.D., Johnston K.C., Hemmen T.M. (2005). TNK in Stroke Investigators A pilot dose-escalation safety study of tenecteplase in acute ischemic stroke. Stroke.

[2] Roger V.L., Go A.S., Lloyd-Jones D.M., Benjamin E.J., Berry J.D., Borden W.B., Bravata D.M., Dai S., Ford E.S., Fox C.S. (2012). American Heart Association Statistics Committee and Stroke Statistics Subcommittee Executive summary: Heart disease and stroke statistics—2012 update: A report from the American Heart Association. Circulation.

[3] Liberatore G.T., Samson A., Bladin C., Schleuning W.-D., Medcalf R.L. (2003). Vampire bat salivary plasminogen activator (desmoteplase): A unique fibrinolytic enzyme that does not promote neurodegeneration. Stroke.

[4] Del Zoppo G.J., Higashida R.T., Furlan A.J., Pessin M.S., Rowley H.A., Gent M. (1998). PROACT: A phase II randomized trial of recombinant pro-urokinase by direct arterial delivery in acute middle cerebral artery stroke. PROACT Investigators. Prolyse in Acute Cerebral Thromboembolism. Stroke.

[5] Qureshi A.I., Ali Z., Suri M.F., Kim S.H., Shatla A.A., Ringer A.J., Lopes D.K., Guterman L.R., Hopkins L.N. (2001). Intra-arterial third-generation recombinant tissue plasminogen activator (reteplase) for acute ischemic stroke. Neurosurgery.

[6] Lapchak P.A., Araujo D.M., Pakola S., Song D., Wei J., Zivin J.A. (2002). Microplasmin: A novel thrombolytic that improves behavioral outcome after embolic strokes in rabbits. Stroke.

[7] Adams H.P., Leclerc J.R., Bluhmki E., Clarke W., Hansen M.D., Hacke W. (2004). Measuring outcomes as a function of baseline severity of ischemic stroke. Cerebrovasc. Dis..

[8] Junghans U., Seitz R.J., Aulich A., Freund H.J., Siebler M. (2001). Bleeding risk of tirofiban, a nonpeptide GPIIb/IIIa platelet receptor antagonist in progressive stroke: An open pilot study. Cerebrovasc. Dis..

[9] Junghans U., Seitz R.J., Ritzl A., Wittsack H.-J., Fink G.R., Freund H.J., Siebler M. (2002). Ischemic brain tissue salvaged from infarction by the GP IIb/IIIa platelet antagonist tirofiban. Neurology.

[10] Nogueira R.G., Schwamm L.H., Hirsch J.A. (2009). Endovascular approaches to acute stroke, part 1: Drugs, devices, and data. AJNR Am. J. Neuroradiol..

[11] Rosamond W., Flegal K., Friday G., Furie K., Go A., Greenlund K., Haase N., Ho M., Howard V., Kissela B. (2007). American Heart Association Statistics Committee and Stroke Statistics Subcommittee Heart disease and stroke statistics—2007 update: A report from the American Heart Association Statistics Committee and Stroke Statistics Subcommittee. Circulation.

[12] Furlan A., Higashida R., Wechsler L., Gent M., Rowley H., Kase C., Pessin M., Ahuja A., Callahan F., Clark W.M. (1999). Intra-arterial prourokinase for acute ischemic stroke. The PROACT II study: A randomized controlled trial. Prolyse in Acute Cerebral Thromboembolism. JAMA.

[13] (1995). Tissue plasminogen activator for acute ischemic stroke. The National Institute of Neurological Disorders and Stroke rt-PA Stroke Study Group. N. Engl. J. Med..

[14] Jahan R., Duckwiler G.R., Kidwell C.S., Sayre J.W., Gobin Y.P., Villablanca J.P., Saver J., Starkman S., Martin N., Viñuela F. (1999). Intraarterial thrombolysis for treatment of acute stroke: experience in 26 patients with long-term follow-up. AJNR Am. J. Neuroradiol..

[15] Caplan L.R., Mohr J.P., Kistler J.P., Koroshetz W. (1997). Should thrombolytic therapy be the first-line treatment for acute ischemic stroke? Thrombolysis—not a panacea for ischemic stroke. N. Engl. J. Med..

[16] Ueda T., Sakaki S., Yuh W.T., Nochide I., Ohta S. (1999). Outcome in acute stroke with successful intra-arterial thrombolysis and predictive value of initial single-photon emission-computed tomography. J. Cereb. Blood Flow Metab..

[17] Saver J.L. (2004). Number needed to treat estimates incorporating effects over the entire range of clinical outcomes: Novel derivation method and application to thrombolytic therapy for acute stroke. Arch. Neurol..

[18] Pillai J.J., Lanzieri C.F., Trinidad S.B., Tarr R.W., Sunshine J.L., Lewin J.S. (2001). Initial angiographic appearance of intracranial vascular occlusions in acute stroke as a predictor of outcome of thrombolysis: Initial experience. Radiology.

[19] Hacke W., Donnan G., Fieschi C., Kaste M., von Kummer R., Broderick J.P., Brott T., Frankel M., Grotta J.C., Haley E.C. (2004). Association of outcome with early stroke treatment: Pooled analysis of ATLANTIS, ECASS, and NINDS rt-PA stroke trials. Lancet.

[20] Arnold M., Schroth G., Nedeltchev K., Loher T., Remonda L., Stepper F., Sturzenegger M., Mattle H.P. (2002). Intra-arterial thrombolysis in 100 patients with acute stroke due to middle cerebral artery occlusion. Stroke.

[21] Rha J.-H., Saver J.L. (2007). The impact of recanalization on ischemic stroke outcome: A meta-analysis. Stroke.

[22] Berlis A., Lutsep H., Barnwell S., Norbash A., Wechsler L., Jungreis C.A., Woolfenden A., Redekop G., Hartmann M., Schumacher M. (2004). Mechanical thrombolysis in acute ischemic stroke with endovascular photoacoustic recanalization. Stroke.

[23] Lewandowski C.A., Frankel M., Tomsick T.A., Broderick J., Frey J., Clark W., Starkman S., Grotta J., Spilker J., Khoury J., Brott T. (1999). Combined intravenous and intra-arterial r-TPA *venous* intra-arterial therapy of acute ischemic stroke: Emergency Management of Stroke (EMS) Bridging Trial. Stroke.

[24] Kleindorfer D., Lindsell C.J., Brass L., Koroshetz W., Broderick J.P. (2008). National US estimates of recombinant tissue plasminogen activator use: ICD-9 codes substantially underestimate. Stroke.

[25] Sundt T.M., Smith H.C., Campbell J.K., Vlietstra R.E., Cucchiara R.F., Stanson A.W. (1980). Transluminal angioplasty for basilar artery stenosis. Mayo Clin. Proc..

[26] Tomsick T., Broderick J., Carrozella J., Khatri P., Hill M., Palesch Y., Khoury J. (2008). Interventional Management of Stroke II Investigators Revascularization results in the Interventional Management of Stroke II trial. AJNR Am. J. Neuroradiol..

[27] IMS Study Investigators (2004). Combined intravenous and intra-arterial recanalization for acute ischemic stroke: The Interventional Management of Stroke Study. Stroke.

[28] IMS II Trial Investigators (2007). The Interventional Management of Stroke (IMS) II Study. Stroke.

[29] Hacke W., Kaste M., Bluhmki E., Brozman M., Dávalos A., Guidetti D., Larrue V., Lees K.R., Medeghri Z., Machnig T. (2008). Thrombolysis with alteplase 3 to 4.5 hours after acute ischemic stroke. N. Engl. J. Med..

[30] Khatri P., Hill M.D., Palesch Y.Y., Spilker J., Jauch E.C., Carrozzella J.A., Demchuk A.M., Martin R., Mauldin P., Dillon C. (2008). Methodology of the Interventional Management of Stroke III Trial. Int. J. Stroke.

[31] Del Zoppo G.J., Saver J.L., Jauch E.C., Adams H.P., American Heart Association Stroke Council (2009). Expansion of the time window for treatment of acute ischemic stroke with intravenous tissue plasminogen activator: A science advisory from the American Heart Association/American Stroke Association. Stroke.

[32] Phatouros C.C., Higashida R.T., Malek A.M., Smith W.S., Mully T.W., DeArmond S.J., Dowd C.F., Halbach V.V. (1999). Endovascular stenting of an acutely thrombosed basilar artery: Technical case report and review of the literature. Neurosurgery.

[33] Kleindorfer D., Kissela B., Schneider A., Woo D., Khoury J., Miller R., Alwell K., Gebel J., Szaflarski J., Pancioli A. (2004). Eligibility for recombinant tissue plasminogen activator in acute ischemic stroke: A population-based study. Stroke.

[34] Nakano S., Iseda T., Yoneyama T., Kawano H., Wakisaka S. (2002). Direct percutaneous transluminal angioplasty for acute middle cerebral artery trunk occlusion: An alternative option to intra-arterial thrombolysis. Stroke.

[35] Nogueira R.G., Schwamm L.H., Buonanno F.S., Koroshetz W.J., Yoo A.J., Rabinov J.D., Pryor J.C., Hirsch J.A. (2008). Low-pressure balloon angioplasty with adjuvant pharmacological therapy in patients with acute ischemic stroke caused by intracranial arterial occlusions. Neuroradiology.

[36] Levy E.I., Siddiqui A.H., Crumlish A., Snyder K.V., Hauck E.F., Fiorella D.J., Hopkins L.N., Mocco J. (2009). First Food and Drug Administration-approved prospective trial of primary intracranial stenting for acute stroke: SARIS (stent-assisted recanalization in acute ischemic stroke). Stroke.

[37] Booth N.A., Robbie L.A., Croll A.M., Bennett B. (1992). Lysis of platelet-rich thrombi: The role of PAI-1. Ann. N. Y. Acad. Sci..

[38] Brommer E.J., Potter van Loon B.J., Rijken D.C., van Bockel J.H. (1992). Composition and susceptibility to thrombolysis of pathological human arterial thrombi. Ann. N. Y. Acad. Sci..

[39] Gobin Y.P., Starkman S., Duckwiler G.R., Grobelny T., Kidwell C.S., Jahan R., Pile-Spellman J., Segal A., Viñuela F., Saver J.L. (2004). MERCI 1: A phase 1 study of Mechanical Embolus Removal in Cerebral Ischemia. Stroke.

[40] Broderick J.P., Palesch Y.Y., Demchuk A.M., Yeatts S.D., Khatri P., Hill M.D., Jauch E.C., Jovin T.G., Yan B., Silver F.L. (2013). Endovascular therapy after intravenous t-PA *versus* t-PA alone for stroke. N. Engl. J. Med..

[41] Xavier A.R., Tiwari A., Kansara A. (2012). Angioplasty and stenting for mechanical thrombectomy in acute ischemic stroke. Neurology.

[42] Roubec M., Kuliha M., Procházka V., Krajca J., Czerny D., Jonszta T., Krajina A., Sanák D., Langová K., Herzig R., Skoloudík D. (2012). A Controlled Trial of Revascularization in Acute Stroke. Radiology.

[43] Smith W.S., Sung G., Starkman S., Saver J.L., Kidwell C.S., Gobin Y.P., Lutsep H.L., Nesbit G.M., Grobelny T., Rymer M.M. (2005). Safety and efficacy of mechanical embolectomy in acute ischemic stroke: Results of the MERCI trial. Stroke.

[44] Smith W.S., Sung G., Saver J., Budzik R., Duckwiler G., Liebeskind D.S., Lutsep H.L., Rymer M.M., Higashida R.T., Starkman S. (2008). Mechanical thrombectomy for acute ischemic stroke: Final results of the Multi MERCI trial. Stroke.

[45] Mayer T.E., Hamann G.F., Brueckmann H. (2002). Mechanical extraction of a basilar-artery embolus with the use of flow reversal and a microbasket. N. Engl. J. Med..

[46] Brekenfeld C., Schroth G., El-Koussy M., Nedeltchev K., Reinert M., Slotboom J., Gralla J. (2008). Mechanical thromboembolectomy for acute ischemic stroke: Comparison of the catch thrombectomy device and the Merci Retriever *in vivo*. Stroke.

[47] Henkes H., Reinartz J., Lowens S., Miloslavski E., Roth C., Reith W., Kühne D. (2006). A device for fast mechanical clot retrieval from intracranial arteries (Phenox clot retriever). Neurocrit. Care.

[48] Schumacher H.C., Meyers P.M., Yavagal D.R., Harel N.Y., Elkind M.S.V., Mohr J.P., Pile-Spellman J. (2003). Endovascular mechanical thrombectomy of an occluded superior division branch of the left MCA for acute cardioembolic stroke. Cardiovasc. Intervent. Radiol..

[49] Henkes H., Lowens S., Preiss H., Reinartz J., Miloslavski E., Kühne D. (2006). A new device for endovascular coil retrieval from intracranial vessels: Alligator retrieval device. AJNR Am. J. Neuroradiol..

[50] Kerber C.W., Wanke I., Bernard J., Woo H.H., Liu M.W., Nelson P.K. (2007). Rapid intracranial clot removal with a new device: The alligator retriever. AJNR Am. J. Neuroradiol..

[51] Bose A., Henkes H., Alfke K., Reith W., Mayer T.E., Berlis A., Branca V., Sit S.P., Penumbra Phase 1 Stroke Trial Investigators (2008). The Penumbra System: A mechanical device for the treatment of acute stroke due to thromboembolism. AJNR Am. J. Neuroradiol..

[52] Penumbra Pivotal Stroke Trial Investigators (2009). The penumbra pivotal stroke trial: Safety and effectiveness of a new generation of mechanical devices for clot removal in intracranial large vessel occlusive disease. Stroke.

[53] Tarr R., Hsu D., Kulcsar Z., Bonvin C., Rufenacht D., Alfke K., Stingele R., Jansen O., Frei D., Bellon R. (2010). The POST trial: initial post-market experience of the Penumbra system: revascularization of large vessel occlusion in acute ischemic stroke in the United States and Europe. J. Neurointerv. Surg..

[54] Ellis J.A., Youngerman B.E., Higashida R.T., Altschul D., Meyers P.M. (2011). Endovascular treatment strategies for acute ischemic stroke. Int. J. Stroke.

[55] A Randomized, Concurrent Controlled Trial to Assess the Safety and Effectiveness of the Separator 3D as a Component of the Penumbra System in the Revascularization of Large Vessel Occlusion in Acute Ischemic Stroke. http://clinicaltrials.gov/show/NCT01584609.

[56] Assess the Penumbra System in the Treatment of Acute Stroke (THERAPY). http://clinicaltrials.gov/show/NCT01429350.

[57] Saver J.L., Jahan R., Levy E.I., Jovin T.G., Baxter B., Nogueira R.G., Clark W., Budzik R., Zaidat O.O., SWIFT Trialists (2012). Solitaire flow restoration device *versus* the Merci Retriever in patients with acute ischaemic stroke (SWIFT): A randomized, parallel-group, non-inferiority trial. Lancet.

[58] Nogueira R.G., Lutsep H.L., Gupta R., Jovin T.G., Albers G.W., Walker G.A., Liebeskind D.S., Smith W.S., TREVO 2 Trialists (2012). Trevo *versus* Merci retrievers for thrombectomy revascularisation of large vessel occlusions in acute ischaemic stroke (TREVO 2): A randomised trial. Lancet.

[59] Mendonça N., Flores A., Pagola J., Rubiera M., Rodríguez-Luna D., Miquel M.A.D., Cardona P., Quesada H., Mora P., Alvarez-Sabín J. (2012). Trevo *versus* Solitaire a Head-to-Head Comparison between Two Heavy Weights of Clot Retrieval. J. Neuroimaging.

[60] Ciccone A., Valvassori L., Nichelatti M., Sgoifo A., Ponzio M., Sterzi R., Boccardi E., SYNTHESIS Expansion Investigators (2013). Endovascular treatment for acute ischemic stroke. N. Engl. J. Med..

[61] Kidwell C.S., Jahan R., Gornbein J., Alger J.R., Nenov V., Ajani Z., Feng L., Meyer B.C., Olson S., Schwamm L.H. (2013). A trial of imaging selection and endovascular treatment for ischemic stroke. N. Engl. J. Med..

